# Adenoma Detection Rates: Does Endoscopic Volume or Provider Specialty Matter More?

**DOI:** 10.3390/jcm14207201

**Published:** 2025-10-13

**Authors:** Jacob Applegarth, Alexander Menning, Michael Tolkacz, Diane Studzinski, Matthew Ziegler

**Affiliations:** Department of Colorectal Surgery, Corewell Health William Beaumont University Hospital, Royal Oak, MI 48073, USA

**Keywords:** adenoma detection rate, colonoscopy, colorectal surgeon

## Abstract

**Background**: Adenoma detection rate (ADR) is a well-established quality indicator for colonoscopy. Previous studies have demonstrated a superior ADR for gastroenterologists versus non-gastroenterologists. However, the number of annual colonoscopies performed by non-gastroenterologists in these studies has been variable and often far fewer than the number performed by gastroenterologists. Our study aims to compare ADR in gastroenterologists and specifically colorectal surgeons of comparable colonoscopy volumes at a single institution. **Methods**: A retrospective chart review of all screening colonoscopies for average-risk patients was performed at a single tertiary care facility, including colonoscopies performed by both gastroenterologists and colorectal surgeons. Univariate analysis was performed using GraphPad Prism v9.3.0. **Results**: No significant difference in overall adenoma detection rates (ADR) was appreciated between gastroenterologists and colorectal surgeons at our institution (36.3% (SD 12.3%) vs. 30.8% (SD 6.7%), respectively, *p* = 0.224). Colorectal surgeons were more likely to have a longer withdrawal time (15.20 min vs. 11.17 min). Gastroenterologists were more likely to collect any specimen during colonoscopy (40.4% vs. 53.6%). However, there was no statistically significant difference in ADR when comparing the top five highest volume colorectal surgeons and the top five highest volume gastroenterologists, and both high-volume groups met recognized benchmarks for male, female, and overall ADR (30%, 20%, 25%). **Conclusions**: Colorectal surgeons removed a similar number of tubulovillous adenomas compared to gastroenterologists. Gastroenterologists tend to remove more polyps overall, including more hyperplastic polyps and sessile serrated adenomas. Despite this, no significant difference in ADR was identified between high-volume colorectal surgeons and gastroenterologists.

## 1. Introduction

Over the past several decades, screening colonoscopy has become an increasingly utilized and important method for detecting and removing adenomatous polyps to reduce the rate of colon cancer. This is especially true as rates of early-onset colorectal cancer rise dramatically in both the United States and Europe [1,2]. In response to increased incidence of early-onset colorectal cancer, recent guidelines from the American Gastroenterological Association now recommend starting colorectal cancer screening at age 45 for average-risk individuals [3]. With the expansion of the eligible screening population, quality measures for colonoscopy have become a popular topic of study and debate. Adenoma detection rate (ADR) is arguably the most important benchmark for quality endoscopy, as higher ADR has been shown to correlate with reduced risk of interval cancer- and colorectal cancer-related mortality [4,5]. For this reason, identifying the patient, technical, or operator factors that impact ADR is of incredible importance.

A minimum overall ADR of 25% (30% for males and 20% for females) [6] has been suggested as a standard quality measure. Several recent studies have shown a significant impact of provider specialty and training background on ADR [7,8], and most suggest a significantly higher ADR for gastroenterologists versus colorectal surgeons, general surgeons, and non-surgeon practitioners. However, these studies often included lower-volume non-gastroenterologist endoscopists, limiting direct comparison with experienced, high-volume colorectal surgeons. More recent literature suggests endoscopic volume and experience may be at least as important as specialty training in determining competency and ADR performance [9,10]. Providers who perform colonoscopy more frequently tend to develop greater technical proficiency and familiarity with polyp detection, which may offset differences attributable to specialty background alone. Therefore, we sought to compare ADRs between gastroenterologists and colorectal surgeons of comparable colonoscopy volume within the same tertiary care institution. By focusing on providers with similar levels of experience and procedural workload, our study aims to clarify the relative contributions of specialty and volume. This comparison will help determine whether high-volume colorectal surgeons achieve ADRs equivalent to their gastroenterologist counterparts, thereby informing ongoing debates about the most critical determinants of endoscopic quality and competency.

## 2. Materials and Methods

We conducted a retrospective cohort study at a single tertiary-care academic medical center for the time period from 1 January 2018 to 31 December 2019. All screening colonoscopies performed by gastroenterologists and colorectal surgeons who met inclusion criteria were identified using institutional endoscopy reporting software and verified through electronic medical record review. This retrospective review received a waver of consent from our Institutional Review Board.

Endoscopists were included if they performed greater than 25 screening colonoscopies during the study period and had minimum 5 years of specialty experience. Data collection was performed via electronic medical record review of endoscopic operative dictations and pathologic specimens. Using this information, individual provider ADR for male patients, female patients, and overall ADR were calculated, as well as average ADR by specialty. Secondary variables and outcomes such as withdrawal time, quality of bowel preparation, patient demographics, and polyp subtypes were also analyzed for their potential impact on ADR.

In an attempt to standardize patient selection as much as possible, this retrospective review focused on first-time screening colonoscopy alone. All asymptomatic patients over the age of 49 at average risk for colorectal cancer who underwent their first screening colonoscopy were included. Patients were excluded for a personal history of polyps, colon cancer, rectal cancer, inflammatory bowel disease (IBD), previous history of colonoscopy, poor or inadequate bowel prep, family history of colon cancer, rectal cancer, or IBD.

In total, 3656 screening colonoscopies performed by 18 gastroenterologists (1702 colonoscopies) and 9 colorectal surgeons (1954 colonoscopies) were reviewed. Statistical analysis was performed using GraphPad Prism (GraphPad Software, Boston, MA, USA, v9.3.0). Continuous variables are reported as mean (SD) or median (IQR), and univariate comparisons made using unpaired *t*-tests or Mann–Whitney ranked sum tests, depending on distribution. Normality of distribution was determined using Shapiro–Wilks test and verified by histogram. Categorical variables are reported as numbers (percentage) and univariate comparisons performed using Χ^2^ or Fisher’s exact test. Linear regression analysis was used to determine the effect of one outlier GI provider with a high number of scopes and a higher ADR, with a curve comparison with and without the outlier provider. Multiple logistic regression was performed to determine the presence or absence of confounding variables and their effect on adenoma detection rates.

## 3. Results

3656 screening colonoscopies performed by eighteen gastroenterologists (1702) and nine colorectal surgeons (1954) as well as characteristics of each endoscopist were reviewed. The average years in practice for gastroenterologists and colorectal surgeons were not statistically different (27.5 years vs. 20.7, *p* = 0.15). Comparison of patient populations showed no significant differences in median age or sex. However, there were statistically significant differences in BMI and race (Table 1).

Univariate analysis demonstrated colorectal surgeons were more likely to have a longer withdrawal time (15.20 min vs. 11.17 min, *p* < 0.0001) and recorded a higher quality of bowel prep (80.2% good or excellent compared to 48.8%, *p* < 0.0001). Gastroenterologists removed any type of polyp significantly more frequently (53.6% vs. 40.4%, *p* < 0.0001), a greater number of polyps that were adenomas (708 vs. 599, *p* < 0.0001), a greater number of hyperplastic polyps (385 vs. 301, *p* < 0.0001), and a greater number of sessile serrated adenomas (78 vs. 45, *p* < 0.0001). There was no difference in number of tubulovillous adenomas removed (53 vs. 52, *p* = 0.41) or cecal intubation rates.

Despite a greater total number of overall adenomas detected in the gastroenterologist provider group, when calculated as adenoma detection rate per provider, no significant difference in ADR between the average gastroenterologist and average colorectal surgeon was found (36.3% (SD 12.3%) vs. 30.8% (SD 6.7%), respectively, *p* = 0.224). There was also no difference in ADR between specialties for male (GI 42.2% (SD 15.0%) vs. CRS 37.0% (SD 8.9%), *p* = 0.347) or female (GI 30.8% (SD 11.3%) vs. CRS 26.2% (SD 9.4%), *p* = 0.306) patients.

This finding held true when examining colorectal surgeons and gastroenterologists who performed a high volume of colonoscopies. The five providers from each group with the greatest number of colonoscopies performed during our study period were compared and no significant difference in overall, male, or female ADR was found (Table 2).

To assess the impact of time spent in clinical practice on ADR, providers with less than 10 years of experience were compared to providers with greater or equal to 10 years of experience. Providers with less than 10 years of experience did not have a significantly different overall, male, or female ADR when compared to providers with greater than 10 years of experience (Table 3).

## 4. Discussion

Adenoma detection rate is an established quality measure for colonoscopy and has been shown to be associated with patient outcomes. Kaminski et al. and Corley et al. showed that higher ADR is associated with lower risk of developing colorectal cancer by disrupting the adenoma to carcinoma sequence [4,5]. Several prior studies have highlighted the superior ADR in gastroenterologists versus non-gastroenterologists. For example, Muthukuru et al. demonstrated that only gastroenterologists met the recognized benchmarks for male, female, and overall ADR [7]. However, prior studies failed to define the overall experience and endoscopic volumes in non-gastroenterologist practitioners. Our study seeks to provide a more balanced comparison by evaluating ADR in relatively high-volume colorectal surgeons and GI physicians in the same institution. In our study, no difference in ADR between gastroenterologists and colorectal surgeons was appreciated, and both specialties exceeded the recognized benchmarks for male, female, and overall ADR (30%, 20%, 25%) [6]. We recognize that our data contains an outlier. One gastroenterologist accounted for 532 total colonoscopies and 297 adenomas detected. Given this, the provider’s overall ADR was 55.8%, male ADR was 65.3%, and female ADR was 45.7%. This provider accounted for 41.9% of all adenomas detected by the entire gastroenterologist group. However, despite this significant outlier being included in our gastroenterologist population, no significant difference in ADR was found when total, male, and female ADR were averaged over the number of providers in each group. Linear regression analysis with ADR as the dependent variable and number of scopes as the independent variable showed no difference in R2 values with and without the outlier provider data.

This finding emphasizes the potential influence of endoscopic volume and experience on ADR rather than specialty training alone. This finding is not otherwise explained by minor differences in BMI and race in the studied patient populations. To demonstrate this, we performed a multivariable regression to identify confounding variables, and no multi-collinearity was found with variance inflation factors between 1.00 and 1.04. Also, the reported difference in BMI of just 1.1 is likely not clinically significant when performing endoscopic evaluation. The colonoscopy requirement during residency and fellowship training has also been a subject of debate, and the number needed to establish competency has not been determined. The current minimum number of colonoscopies required by the ACGME for both gastroenterology and colorectal surgery fellowships is 140. Several studies have suggested 250 colonoscopies as the requirement to assess competency, based on cecal intubation success rate and other recent AGA guidelines [9]. However, other reports suggest a number as high as 400 to establish proficiency [10]. In our study, we were not able to determine the number of colonoscopies performed or years in practice necessary to achieve competency. Our data showed no difference in those practicing 10 years or more compared to less than 10 years; however, our cohort only had two physicians that had been practicing less than 10 years duration.

Further investigation to determine the impact of endoscopy volume and experience is warranted. Continued efforts to improve the quality of training will be necessary to improve ADR, and recent training models such as entrustable professional activities (EPA), proficiency-based examination, and simulation education may prove to be beneficial.

Our study has several limitations. The data were reviewed retrospectively, whereas a prospective data evaluation would likely provide a higher quality comparison between practitioner groups. Another limitation is the low number of endoscopists in practice for less than 10 years, which makes it difficult to determine the impact of experience on competency and ADR. Also, the number of colonoscopies evaluated in this study was significantly higher for colorectal surgeons compared to gastroenterologists, and this is due to the large volume of colonoscopies performed by our gastroenterologists at outpatient endoscopy centers. We recognized that different practice settings could introduce bias as certain patients are selected to receive their care at the inpatient care center. However, we feel that our inclusion and exclusion criteria were robust enough to mitigate this bias.

## 5. Conclusions

Colorectal surgeons removed a similar number of tubulovillous adenomas compared to gastroenterologists. Gastroenterologists tend to remove more polyps overall, including more hyperplastic polyps and sessile serrated adenomas. Despite this, our study showed no significant difference in ADR between high-volume, highly experienced gastroenterologists and colorectal surgeons. Further investigation to determine competency and improve the quality of training and practice is warranted.

## Figures and Tables

**Table 1:** Patient Population Demographics.

	Total Population (n = 3656)	Gastroenterologist (GI) (n = 1702)	Colorectal Surgeon (CRS) (n = 1954)	*p* Value
Age, years	58.7 (49.5, 90.6)	58.2 (52.2, 65.4)	59.0 (52.6, 64.8)	0.505 ^a^
Sex, female	1881 (51.4)	1028 (52.6)	853 (50.1)	0.133 ^b^
BMI, kg/m^2^	27.4 (24.3, 31.7)	28.0 (24.7, 32.1)	26.9 (24.0, 31.2)	**<0.0001** ^a^
Race				**<0.0001** ^b,c^
Caucasian/White	2475 (67.7)	1043 (61.3)	1432 (73.3)	
African American/Black	762 (20.9)	425 (25.0)	337 (17.3)	
Asian	180 (4.9)	113 (6.6)	67 (3.4)	
Other	208 (5.7)	106 (6.2)	102 (5.2)	
Prefer not to answer	29 (0.8)	14 (0.8)	15 (0.8)	
Ethnicity				257 ^b,c^
Non-Hispanic/Latino	3122 (85.5)	1440 (84.7)	1682 (86.2)	
Hispanic/Latino	38 (1.0)	14 (0.8)	24 (1.2)	
Arab/Middle Eastern	112 (3.1)	60 (3.5)	52 (2.7)	
Other	222 (6.0)	107 (6.3)	115 (5.9)	
Unknown/prefer not to answer	159 (4.4)	80 (0.8)	79 (4.0)	

Abbreviations: colorectal surgeon (CRS), gastroenterologist (GI), interquartile range (IQR), kilogram (kg), meter (m). Continuous data shown as median (IQR), categorical data shown as n (%); *p* values for comparison of GI and CRS; *p* values < 0.05 bolded. ^a^ Mann–Whitney ranked sum test. ^b^ Χ^2^ or Fisher’s exact test. ^c^ Race comparison minus Unknown/prefer not to answer; ethnicity comparison minus Other and Unknown/Prefer not to answer.

**Table 2:** Adenoma Detection Rate for High Volume Providers.

	Top Five High Volume GIs	Top Five High Volume CRS	*p* Value
Overall ADR, %	41.2 (11.0)	30.6 (6.0)	0.096
Female ADR, %	34.6 (9.1)	25.3 (6.4)	0.099
Male ADR, %	48.1 (13.3)	38.1 (10.5)	0.223
Total number of colonoscopies	308.8 (72.7)	230.4 (197.2)	0.429

Abbreviations: adenoma detection rate (ADR), colorectal surgeon (CRS), gastroenterologist (GI). standard deviation (SD). Continuous data shown as mean (SD), all unpaired *t* tests.

**Table 3:** Provider Years of Clinical Experience and Adenoma Detection Rate.

	6–10 Years in Practice (n = 2)	>10 Years in Practice (n = 22)	*p* Value
Overall ADR, %	32.7 (25.8, 39.6)	35.6 (29.4, 44.7)	0.652
Female ADR, %	24.5 (12.5, 36.4)	30.5 (23.5, 39.2)	0.518
Male ADR, %	46.9 (40.0, 53.8)	40.7 (33.1, 53.6)	0.587
Total number of colonoscopies	121.5 (31.0, 212.0)	230.4 (197.2)	0.866
Years in practice	6.5 (6.0, 7.0)	25.5 (19.8, 33.5)	**0.004**

Abbreviations: adenoma detection rate (ADR), interquartile range (IQR). Continuous data shown as median (IQR), all Mann–Whitney ranked sum tests, *p* values < 0.05 bolded.

## Data Availability

The data are not publicly available due to privacy restrictions and can be obtained by contacting the corresponding author.
